# *Glycyrrhiza glabra* (Licorice): A Comprehensive Review on Its Phytochemistry, Biological Activities, Clinical Evidence and Toxicology

**DOI:** 10.3390/plants10122751

**Published:** 2021-12-14

**Authors:** Shadma Wahab, Sivakumar Annadurai, Shahabe Saquib Abullais, Gotam Das, Wasim Ahmad, Md Faruque Ahmad, Geetha Kandasamy, Rajalakshimi Vasudevan, Md Sajid Ali, Mohd Amir

**Affiliations:** 1 Department of Pharmacognosy, College of Pharmacy, King Khalid University, Abha 61421, Saudi Arabia; sannadurai@kku.edu.sa; 2 Department of Periodontics and Community Dental Sciences, College of Dentistry, King Khalid University, Abha 61421, Saudi Arabia; drsaquib24@gmail.com; 3 Department of Prosthodontics, College of Dentistry, King Khalid University, Abha 61421, Saudi Arabia; gmenghwar@kku.edu.sa; 4 Department of Pharmacy, Mohammed Al-Mana College for Medical Sciences, Safaa, Dammam 34222, Saudi Arabia; wasimahmadansari@yahoo.com; 5 Department of Clinical Nutrition, College of Applied Medical Sciences, Jazan University, Jazan 45142, Saudi Arabia; mfahmad@jazanu.edu.sa; 6 Department of Clinical Pharmacy, College of Pharmacy, King Khalid University, Abha 61421, Saudi Arabia; glakshmi@kku.edu.sa; 7 Department of Pharmacology, College of Pharmacy, King Khalid University, Abha 61421, Saudi Arabia; raja@kku.edu.sa; 8 Department of Pharmaceutics, College of Pharmacy, Jazan University, Jazan 45142, Saudi Arabia; mdsajidaali@gmail.com; 9 Department of Natural Products and Alternative Medicines, College of Clinical Pharmacy, Imam Abdulrahman Bin Faisal University, P.O. Box 1982, Dammam 31441, Saudi Arabia; matahmad@iau.edu.sa

**Keywords:** *Glycyrrhiza glabra*, phytochemistry, respiratory infection, anticancer, hepatoprotective, cardiovascular

## Abstract

There are more than 30 species of Glycyrrhiza genus extensively spread worldwide. It was the most prescribed herb in Ancient Egyptian, Roman, Greek, East China, and the West from the Former Han era. There are various beneficial effects of licorice root extracts, such as treating throat infections, tuberculosis, respiratory, liver diseases, antibacterial, anti-inflammatory, and immunodeficiency. On the other hand, traditional medicines are getting the attraction to treat many diseases. Therefore, it is vital to screen the medicinal plants to find the potential of new compounds to treat chronic diseases such as respiratory, cardiovascular, anticancer, hepatoprotective, etc. This work comprehensively reviews ethnopharmacological uses, phytochemistry, biological activities, clinical evidence, and the toxicology of licorice, which will serve as a resource for future clinical and fundamental studies. An attempt has been made to establish the pharmacological effect of licorice in different diseases. In addition, the focus of this review article is on the molecular mechanism of licorice extracts and their four flavonoids (isoliquiritigenin, liquiritigenin, lichalocone, and glabridin) pharmacologic activities. Licorice could be a natural alternative for current therapy to exterminate new emerging disorders with mild side effects. This review will provide systematic insights into this ancient drug for further development and clinical use.

## 1. Introduction

Nature has always been a great source of therapeutic substances, delivering us various medicinal plants that produce valuable phytochemicals. Licorice is scientifically known *as Glycyrrhiza glabra* and belongs to the Leguminosae family. *G. glabra* is an ayurvedic herb that is frequently utilized. This medicinal plant is found throughout Asia as well as in areas of Europe [1]. Licorice is thought to have originated in Iraq [2]. *G. glabra*, the most extensively dispersed species, is found in Italy, Spain, Turkey, the Caucasus, western China, and Central Asia. In contrast, *G. uralensis* is located in Central Asia to China and Mongolia [3]. It is grown commercially in Italy, Spain, Greece, France, Iran, Iraq, Turkey, Turkmenistan, Uzbekistan, Syria, Afghanistan, Azerbaijan, India, China, the United States, and England [4,5]. Licorice is one of the most commercially valuable plants globally, having a wide range of uses in tobacco, cosmetics, the food industry, and pharmaceuticals [6]. Phytochemical and pharmaceutical analysis has been extensively explored thoroughly of licorice [3,7,8,9,10]. In traditional Chinese medicine (TCM), *Glycyrrhiza glabra* is considered an “essential herbal medication.” According to a traditional Chinese medicine belief, “nine out of ten formulae contain licorice,” and licorice is one of the most effective herbal medicines for reducing toxicity and increasing the efficacy of other herbal medicines when used together. It may also be a health food product and natural sweetener because it is a “medicine food homology” herbal medication [11]. *Glycyrrhiza glabra*, one of the about 30 kinds of licorice, is one of the most widely utilized species in feed and food [12]. Amino acids, proteins, simple sugars, polysaccharides, mineral salts, pectin, starches, sterols, gums, and resins are all found in licorice [13].

Isoliquiritigenin (2’,4’,4-trihydroxychalcone, ISL) extracted from licorice root has a chalcone structure that exhibits a strong anticancer effect. Glycyrrhizin, glycyrrhizinic acid, isoliquiritin, and glycyrrhizic acid are other main chemicals in this plant with anti-atherogenic, anti-cancer, anti-diabetic, anti-microbial, antispasmodic, anti-inflammatory, and anti-asthmatic properties [14]. Licorice has also been documented to help with weariness and debilitation in China. In addition, licorice acts as an anti-inflammatory, reducing allergic responses and preventing liver damage. According to the World Health Organization, licorice is used as a demulcent for sore throats and an expectorant for bronchial catarrh and coughs [15]. There have been no reports of potentially toxic compounds from the taxa that have been studied so far. However, some adverse consequences are recognized, such as using high dosages over a prolonged period, resulting in serious illnesses. Nevertheless, the plant may be used for a medicinal purpose in small dosages for significant ailments, and there are no known side effects.

In the current review, many chemical constituents of licorice were studied for their substantial pharmacological properties, such as anticancer, antibacterial, anti-inflammatory, cardioprotective, hepatoprotective, against respiratory infection, and many more. Based on the literature, licorice has fascinated the attention of many researchers in current years, and they are dedicated to uncovering its active constituents and their mechanism of action. Flavonoids of licorice are one of licorice stem and root extracts, and they have shown many promising biological activities. This review analyzed ethnopharmacological uses, phytochemical, biological activities, clinical evidence, and toxicology of licorice which will serve as a resource for future clinical and fundamental studies. In addition, the focus of this review article is on the molecular mechanism of licorice extracts and their four flavonoids (isoliquiritigenin, liquiritigenin, lichalocone, and glabridin) pharmacologic activities. Licorice could be a natural alternative for current therapy to exterminate new emerging disorders with mild side effects. This review will provide systematic insights into this ancient drug for further development and clinical use.

## 2. Biodiversity of Licorice

**Figure 1:**
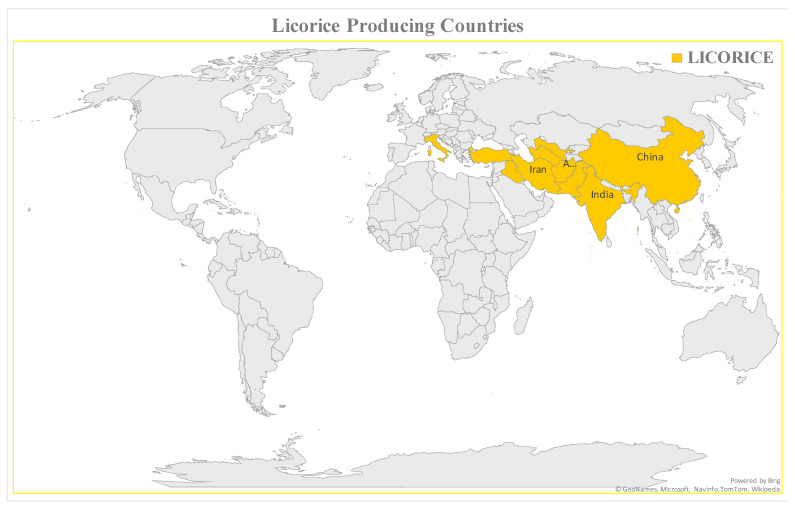
Licorice cultivated countries on the world map.

There are more than 30 species of Glycyrrhiza genus extensively spread worldwide [16]. Licorice has been planted since the 16th century in Europe. It was the most prescribed herb in Ancient Egyptian, Roman, Greek, East China, and the West from the Former Han era [17]. Various species of licorice are cultivated in Europe, the USA, South-Western Asia and Central Africa, the Middle East, Afghanistan, and the North part of India. In addition, England, Spain, Iraq, Turkey, China, and Sicily commercially cultivate licorice [18]. Other countries which are also producing the licorice are Pakistan, Azerbaijan, Turkmenistan, and Uzbekistan. On the world map, licorice-producing areas are shown in Figure 1.

## 3. Secondary Metabolites of Licorice

Phytochemistry is the discipline of science that deals with plant-derived phytochemicals [19]. This section of the article deals with various secondary metabolites found in licorice. Secondary plant metabolites are divided into multiple groups based on their chemical structures. Therefore, it is crucial to explore the main pharmacological activities of various secondary metabolites of licorice such as flavanones, coumarins, chalcones, isoflavones, and many more are contained by triterpenoid saponins and phenolic compounds of licorice [20]. Around 400 total compounds have been isolated from licorice, including approximately 300 flavonoid compounds [21]. The main active constituents of glycyrrhizin, glycyrrhetinic acid, and their derivatives are triterpenoid [22,23,24,25]. Glycyrrhizin can be converted to glycyrrhetinic acid through the metabolic mechanism in humans. Hence, the pharmacological results of glycyrrhetinic acid are the same as glycyrrhizin [26]. Glabridin is the most abundant isoflavone, accounting for 0.08 percent to 0.35 percent of the dry weight of the roots [27]. One molecule of 18-glycyrrhetinic acid and two molecules of glucuronic acid make up *Glycyrrhiza glabra* (18-glycyrrhetinic acid-3-O—D- glucuronopyranosyl-(1 2)—D- glucuronide) [28,29]. Phytoconstituents of *Glycyrrhiza glabra* and their mechanism of action are exhibited in Table 1.

## 4. Ethnobotany of Licorice

Licorice is grown extensively throughout the Middle East, Asia, and Europe. Glycyrrhizin, the main ingredient in licorice, is also known as glycyrrhizinic acid, and it is roughly 50 times sweeter than sucrose. It is commonly used as a natural sweetener and herbal medicine. Licorice is mainly utilized in non-medicinal forms in Western nations, including herbal teas, soft drinks, and tobacco products. It has been used in Chinese herbal medicine for 1000 years. In China, licorice is considered a “vital natural medication.” The Chinese have utilized licorice to tonify the heart and spleen’s qi (life force) since 25 A.D. It is also helpful to relieve pain, phlegm, spasms, cough, and dyspnea. Several pieces of research on licorice have been done since the 1960s. Glycyrrhizin, glycyrrhizinic acid, isoliquiritin, and glycyrrhizic acid are chemicals found in this plant with anti-atherogenic, anti-cancer, anti-diabetic, anti-microbial, antispasmodic, anti-inflammatory, and anti-asthmatic properties [51,52,53,54]. This herb can be used to treat dementia, cognitive impairment, and Alzheimer’s disease. Licorice roots, extracts, and active ingredients like isoflavonoids, flavonoids, and glycyrrhizic acid have demonstrated efficacy in regulating respiratory functions, immunoregulation, antineoplastic action, antiinflammation, gastroenteric protection, and hepatoprotection [15,55], making it the most crucial herb and the focus of herbal medicine research.

### Licorice Traditional and Modern Preparations

Many traditional licorice formulations are now patented in China, such as oral licorice solution is prescribed to treat bronchitis, colds, cough, and upper respiratory infections. Fuzi lizhong tablet treats epigastric crymodynia, spleen and stomach problems, vomiting, diarrhea, and cold hands and feet. Also, Colds, fevers, headaches, dry mouth, cough, and sore throat are treated with Yinqiao Powder. Spleen and stomach qi insufficiency, loose feces, and lack of appetite treated with Sijunzi Granule [11,56,57]. In Indian Traditional Medicine Systems (Ayurveda and Siddha), *Glycyrrhiza glabra* is used as a purgative, ulcer-protecting, anti-tussive, and expectorant medication. Glycyrrhiza root extract is utilised as an eye drop to treat conjunctivitis in India. An example of some Indian pharmaceutical formulations containing *Glycyrrhiza glabra* is GutGard^®^ has more than 10% flavonoid content used as an antioxidant and supports a normal and healthy gastrointestinal tract. Health Aid Licorice is used for dry cough and promotes clear and comfortable breathing. Chewable DGL Licorice tablets are utilised as health supplements. Himalaya company clear complexion whitening face wash contains pomegranate, saffron, licorice, and white dammer, removing dark spots, cleaning, and clarifying impurities. Furthermore, Solaray Licorice 450 mg-100 capsules are used as a nutraceutical product [58].

*Glycyrrhiza glabra*, radix is utilized like an excipient in herbal teas and as an extract in other medical products in several European nations such as Germany, Austria, Czech Republic, Netherlands, Austria, and Norway). A Liquiritiae radix soft extract has been on the market in Germany as traditional medicinal medicine, orally used to promote gastric function, at least since 1976. According to the German Standard Marketing Authorisation procedure, numerous combination products, primarily with Hederae helicis folium and Thymi herba, are on the market in the form of herbal teas. In France, two combos are available; first, glycyrrhiza extract in conjunction with levomenthol, which is used to ease throat irritations. The second one is an herbal tea with liquiritiae radix and Melissa, traditionally utilized to aid digestion [59].

Licorice comes in a variety of forms to treat various diseases, including dried root infusions and decoctions. The licorice doses vary depending upon the type of preparation and health problem. Three times a day, licorice 1.0 to 5.0 g of dried root powder is recommended for asthma. The dose is 2 to 5 mL three times a day if using a licorice tincture (1:5 strength is usual). Additionally, standardized licorice extract dose of 250 to 300 mg three times a day (containing 20% glycyrrhizic acid) is recommended for asthma. In the case of gastroesophageal reflux disease, two 380-mg tablets of deglycyrrhizinated licorice (DGL) should be taken before meals [56].

Industrial uses of licorice include a sweetener in cigarettes, chewing tobacco, chocolate candy, smoking mixes, and chewing gum. In cosmetology, licorice is used as a depigmenting agent. Health product containing glycyrrhizinic acid includes licorice tea, flavoured diet gum, cough mixtures, throat pearls, and herbal cough mixtures. Alcoholic drinks contain all types of licorice root extracts; also, it is included in chewing tobacco. In confectionery, licorice cakes, bricks, sticks, toffee, balls, bars, and gums are used. Glycyrrhiza is frequently utilized in the manufacture of biomass, bioenergy, and pulp. Furthermore, licorice may be converted into a popular livestock feed [60]. “Glabridin-40” is a glabridin-rich extract of *G. glabra* recognized in the International Nomenclature of Cosmetic Ingredients, which is employed rationally in cosmetics compositions [61]. M&F Worldwide is the world’s top maker of licorice goods, producing more than 70% of the global licorice flavors supplied to end-users. Mafco Worldwide uses licorice root to make a variety of licorice products and distributed around the world to confectioners, cosmetic companies, flavoring and masking agents to food processors, and pharmaceutical companies [62].

## 5. Effect of Licorice in Different Diseases

### 5.1. Anticancer Effect of Licorice

Cancer is a prominent root of fatality and ailments worldwide [63,64,65]. It is now the world’s second most significant cause of mortality (9.6 million) [66]. Cancer cases are forecasted to move up speedily in coming decades due to advancements in the style of living and changes in demeanor such as obesity, smoking, physical inactivity, and reproductive pattern. In addition, economic development and urbanization are a few causes of various cancers [67]. Extensive use of chemotherapy and drug-resistant at an alarming rate is the cause of tumors failing to respond [68]. Therefore, natural products are getting popular as anticancer agents to treat drug-resistant malignancies due to their lack of adverse effects, high antitumor property, low toxicity, and specific multi-targeting activities.

Licorice is a primarily used Chinese herb, is generally practised in the medication of liver, gastric, and respiratory disorders, and mitigate the toxicity provoked by other herbs in TCM. Licorice is one of the most extensively examined herbal drugs. It has solid pharmacological properties [12,69]. The name “flavonoids” refers to an arrangement of microscopic molecules having a benzene ring related to a pyrone ring with an identical structure. Flavonoids extensively exist in the natural world and are generally present as O- or C-linked -glycosides in herbs. The majority of the flavonoids can be segregated into many groups, such as flavonoids, flavones, and isoflavonoids [70]. Flavonoid has been classified in to nine groups such as flavan-3-ol, dihydrochalcone, chalcone, isoflavanone, isoflavone, flavanonol, flavanone, flavonol, flavone. In addition, more than three hundred flavonoid monomeric compositions were singled out in licorice in the last ten years. A few are distinct constituents of licorice that are extracted only from licorice, such as various licorice chalcones (isoliquiritin episode, licochalcone C, licochalcone D, etc.), flavanonol and isoflavone (glicoricone, licoricone, isoangustone A, glisoflavone, etc.) [71]. Flavonoids are the main effective constituents extracted from rhizomes and the roots of licorice. Several studies have exhibited that these components contain properties that suppress the expansion of cells derived from different cancers [72,73], such as gastric cancer [74,75], breast cancer [76], and melanoma [77,78]. The review contributes substantial indication that confirms the inherent anticancer potential of licorice and its constituents and provides the base for future studies of their mechanism of action.

One of the most active components in the roots of Glycyrrhiza is isoliquiritigenin (ISL) that has shown direct inhibitory impact on malignancies such as cervical, hepatoma, colon, breast, prostate, and other types of cancers. ISL can also inhibit multistage carcinogenesis processes by promoting progression, formation, and migration by promoting cell cycle, apoptosis, autophagy, anti-angiogenesis, and other actions [79]. In addition, licorice’s anticancer, anti-inflammatory, antioxidant, and antibacterial activities have been related to various human health benefits in pharmacological investigations [80].

**Figure 2:**
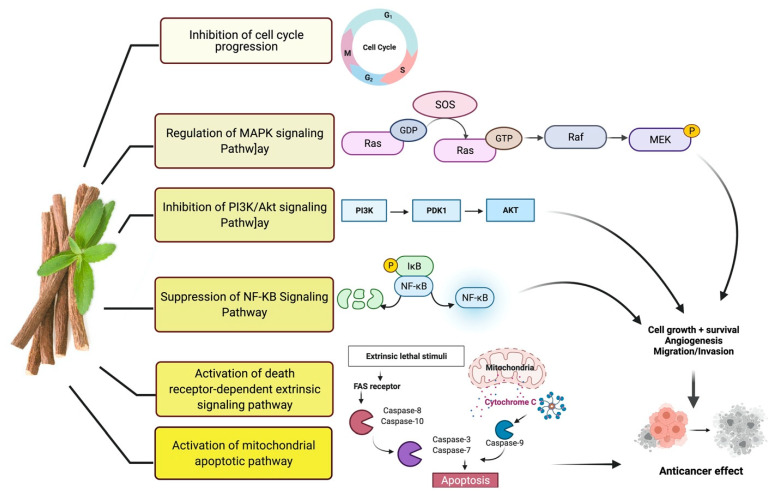
Possible anticancer mechanism of action of *Glycyrrhiza glabra.*

Studies have shown that twelve licorice flavonoids inhibited cancer cell proliferation by suppressing the cell cycle at different phases and causing apoptosis. The proposed anticancer effect of *Glycyrrhiza glabra* has been illustrated in Figure 2. Licochalcone A (LA), a flavonoid found in licorice, has anti-cancer properties. Licochalcone A showed anticancer activity by activating autophagy by upregulation of autophagosome forming LC3-II protein [81]. In another study Licochalcone A upregulated LC3-II signaling and downregulate the PI3K/RAC-α serine-threonine-protein kinase (Akt)/mammalian target of rapamycin (mTOR) signaling [82]. Licochalcone A stops cell cycle advancement at the G1/S and G2/M stages. Its mechanism includes lessening the protein levels of cyclin and mRNA and cyclins and cyclin-dependent kinase (CDK), for instance, Cyclin B1 and CDK1 [82,83,84,85,86,87]. Licochalcone A related mechanism was assessed via T (CD3e (+)), B (CD45R/B220 (+)), and RNA-seq cells in the spleen and whole blood were quantified via flow cytometry. According to the MWM test results, LA enhanced cognitive function and increased CBF levels in treated mice. Thus, LA has the potential to improve cognition by regulating the immune system [88]. In a study, ethanolic and water extracts were analysed the anticancer activity of licorice. Ethanolic extract of licorice has been validated as an anticancer agent to treat various cancers such as breast, colon, and liver. The ethanolic extract has exhibited substantial anti-breast cancer activity and anti-hepatic cancer activity at 100 g/mL and 16.1 g/mL, but no effect on colon cancer at 100 g/mL [89]. Another study examined the impact of ethyl acetate extracts on licorice’s dry leaves and roots and its health uses. LC-MS-MS was used to quantify forty bioactive chemicals, and its significant differences between the extracts were discovered. All the data show that the fresh root of the examined plant has pharmacological potential. This study concluded that licorice could limit and treat sickness due to oxidative stress, such as cancer [90]. Glabridin another important flavonoid of *G. glabra* showed anticancer activity by reducing the expression level of p-epidermal growth factor receptor-like as p-AKT, p-ERK1/2, cyclin D1, and so on [91]. All the above-discussed studies support the use of licorice to treat various types of cancer.

#### 5.1.1. Effect on Human Cervical Cancer

The fourth most prevalent cancer in women is cervical cancer worldwide, and it kills about a quarter of a million individuals every year. Several research has also looked at ISL’s anti-cervical cancer properties. ISL shows their anticancer activity by inducing intrinsic apoptosis in HeLa cells. Apoptosis in HeLa cells was caused by oxidative stressors, mitochondrion-dependent signaling pathways, and estrogen receptor stress-triggered signaling pathways. Isoliquiritigenin therapy reduced cell growth and enhanced apoptosis in HeLa cells and cancer U14 cells. In an in vivo experiment, ISL increased anticancer efficacy and reduced micronucleus production of DNA strand breaks in KM mice carrying U14 when given in conjunction with cyclophosphamide; results concluded that ISL could be an encouraging option to limit and treat cervical cancer [92].

#### 5.1.2. ISL Effects on Breast Cancer

Worldwide in women, breast cancer is one of the leading causes of death. Despite early diagnosis and multimodal therapies improvements, recurrence ratios and rates of breast cancer continue to be poor, particularly in industrialized nations. Breast cancer is the cause of 15% of all deaths due to cancer, and it took the lives of 627,000 women in 2018 [93]. Multiple causes of breast cancer are genetic and epigenetic aberrations, tumor microenvironment, cancer stem cells (CSCs), and many more. Most breast cancer is caused due to the expression of the ER-positive type of estrogen. Two-thirds of all breast cancers are due to estrogen for tumor development. In ER-positive breast cancer, hormone treatment or aromatase inhibitors are often used. Aromatase inhibitors may limit the conversion of testosterone to estrogen, which may have tumor-suppressing effects. Many previous studies have suggested that ISL may act as an aromatase inhibitor due to its mechanism of action [94,95,96,97]. Current therapies for breast cancers are surgical exclusion, radiation, chemotherapy, and medicine such as doxorubicin, paclitaxel, epirubicin, cisplatin, and 5-FU (5-fluorouracil). However, medication resistance and significant adverse effects of these therapy approaches have severely limited their therapeutic potential. As a result, new and safer chemotherapeutic methods are required [95,96].

Vascular endothelial growth factor receptor-2 (VEGFR-2)/vascular endothelial growth factor (VEGF) might be inhibited by naturally isoliquiritigenin to treat breast cancer. ISL suppressed VEGF expression in breast cancer cells by enhancing HIF-1 proteasome degradation and interacted directly with VEGFR-2 to reduce its kinase activity. Treatment of breast cancer with ISL has shown that it suppressed the development of breast cancer and neoangiogenesis. In addition, ISL increased the apoptosis ratio and inhibited VEGF/VEGFR-2 with minimal side effects. Therefore, it’s possible that ISL reduced VEGF production in breast cancer cells by increasing HIF-1 proteasome degradation and interacted directly with VEGFR2 to limit its kinase activity [98]. Lin et al. (2017) have shown that ISL combination with doxorubicin or alone is effective in treating cancer cells. Furthermore, it is very active in sensitizing doxorubicin-resistant cancer cells due to cancer cell death [99]. Various studies have validated that ISL suppresses cancer cell proliferation by inducing autophagy and apoptosis and enhancing chemosensitivity [35,100,101,102].

#### 5.1.3. Effect on Hepatoma Cancer

The most common primary malignant tumor of the liver in adults is hepatocellular carcinoma (HCC), also known as hepatoma. Traditional herbal remedies, like licorice, have long been used to treat and prevent HCC. ISL, a licorice-derived chemical, has recently been utilized to treat hepatoma. In terms of hepatoma cancer cells, ISL exhibits the following anti-hepatoma cancer properties. First, Cuendet et al. (2010) proposed that ISL showed chemoprevention effect in murine hepatoma cells by inducing phase II enzymes in the liver, such as quinone reductase-1 and glutathione and glutathione S-transferase [103]. Second, ISL was discovered to be a monofunctional inducer with the capacity to activate quinone reductase in wild-type Hepa 1c1c7 cells, lowering the risk of cancer [102]. Third, ISL might act as a natural antioxidant in human hepatoma cells, reducing ROS (HepG2) [101]. Fourth, ISL inhibited cell growth in HepG2 cells by halting the G2/M transition and programmed cell death. Another method might be that ISL activates p53, which subsequently activates p21/WAF1, Fas/apolipoprotein-1 receptor, Fas ligand, Bax, and NOXA [100].

#### 5.1.4. Effect on Colon Cancer (CC)

Colon cancer (CC) at third to give rise to cancer-related deaths. If found late, the chance of survival is only 10%. CC is quite frequent in the elderly, and it is a severe public health concern in every country [104]. COX-2 is linked to colon cancer. Selective COX-2 inhibitors and nonselective NSAIDs lessen the CC burden; their cardiovascular and gastrointestinal adverse effects restrict their curative use. A study was conducted by Zhang et al. to exhibit the inhibition of the enzyme 11β–hydroxysteroid dehydrogenase type II (11βHSD2). Results of the study have shown that it lessens tumor growth, the activity of tumor COX-2, and metastasis by enhancing the glucocorticoid-mediated suppression of the COX-2 signaling pathway without any side effects of COX-2 inhibitors and NSAIDs. The findings of these researchers suggested that inhibition of 11βHSD2 may be the potential treatment alternative in CC, and further studies could be done [105,106]. Glycyrrhetinic acid (GE) and flavonoids are recognised to suppress 1βHSD2. In these circumstances, cortisol can inhibit COX-2 expression through the glucocorticoid receptor to inhibit tumorigenesis [106]. A mouse xenograft model investigated the effectiveness of licorice extract antitumor activity alone and in combination with cisplatin and its protective impact against cisplatin-induced toxicity. This model showed that administering licorice extract to BALB/C mice implanted with CT-26 colon cancer cells significantly reduced tumor development. Furthermore, licorice extract with cisplatin reduced cisplatin’s therapeutic effectiveness while substantially increasing the anticancer activity of the licorice extract. Moreover, licorice extract treatment significantly decreased cisplatin-induced oxidative stress. Thus, in combination, licorice extract suppresses the development of mice colon cancer without causing any side effects and lowers the toxicity caused by cisplatin. As a result, licorice extract might be used as an anticancer and chemo preventive agent. On the other hand, patients receiving cisplatin treatment should avoid using licorice extract supplements [107]. A study was conducted to examine the anticancer properties of licorice extracts and ginger and the synergistic effects of management of taken together. In vitro and in vivo, the study found that a synergetic combination of licorice and ginger extracts can reduce colon cancer development, enhance CTL infiltration to the tumor site, and boost apoptosis. As a result, future clinical trials can employ the created combination [108].

Isoliquiritigenin was found to be an effective antioxidant agent to limit and treat 1,2-dimethylhydrazine-induced CC [35]. ISL could affect the resistance of TRAIL (tumor necrosis factor-related apoptosis-inducing ligand) in HT29 cells of CC, principally by enhancing the supply of dearth receptors 5 and protein amid TRAIL receptors, along with the chemopreventive activity of ISL medication joined with TRAIL [103]. ISL impacts not just the metabolic system but also tumor development by inducing apoptosis and autophagy. ISL reduced tumor development by downregulating the anti-apoptotic proteins Bcl-2 and Bcl-x(L), which were halted in G2, according to Auyeung et al. (2010) [109]. Furthermore, ISL significantly lessens NO and PGE2 in human and mouse CC cells [110]. ISL has been recognized as a potential MDR(multidrug-resistant) modulator candidate because of its potential to curb the expression of the caspase 8, caspase 3, AhR, ABCB1, ABCC1 GSTP1, and CYP1A1 genes in colon-MDR cells [111]. Thus, it can be concluded that ISL prohibits tumors by deregulating NO, ROS genesis, PGE-2, COX-2, and NF-кβ activity.

#### 5.1.5. Effect on Pancreatic Cancer

Pancreatic cancer kills thousands of individuals every year throughout the world. It continues to be a dangerous illness that requires immediate care due to its low overall survival rate. Early diagnosis and efficient treatments are two of the biggest hurdles in the fight against cancer [112]. This section reviewed strategies concerning pancreatic cancer therapy and thoroughly described the most recent developments using licorice as a natural treatment. It is licorice’s significant component, and this compound induced ROS in Rh30 and RD rhabdomyosarcoma (RMS) cells. CF (3) DODA-Me inhibited invasion, proliferation, and triggered death in RMS cells, and these effects were mitigated by cotreatment with the antioxidant glutathione, demonstrating ROS’ anticancer action in RMS cells. The impact of CF(3)DODA-Me on cell and tumor development highlights RMS cells’ sensitivity to ROS inducers and their potential clinical implications for treating this devastating illness [113].

The c-Jun N-terminal kinases (JNK) are involved in various physiologic processes triggered by mixed stress signals. In diabetes, Parkinson’s illness, and cancer, each JNK protein has different functions. Licochalcone A, a major phenolic component isolated from licorice root, inhibited JNK1 activity in vitro but had minimal influence on JNK2 activity. The simulation model demonstrated that licochalcone A inhibits JNK1’s affinity for ATP binding more than JNK2. In vitro and in vivo, licochalcone A was not suppressed JNK2-mediated and was suppressed JNK1-mediated. JNK1 was exhibited significantly expressed in pancreatic cancer cell lines compared to normal cell lines. The suppression caused apoptosis and G (1) phase arrest. Treatment with licochalcone A or knocking down JNK1 expression suppressed colony formation and pancreatic cancer cell proliferation in cancer cell lines. These findings indicate that licochalcone A is a JNK1 inhibitor that is selective. Therefore, licochalcone A might have precautionary or curative potential against pancreatic cancer [114]. The antiproliferative efficacy and synthesis of 3-O-ether derivatives of glycyrrhetic acid were reported in another investigation. The cytotoxicity of the produced compounds was examined in human pancreatic cancer cell lines (MIAPaCa-2). Compound 2,6-dichlorobenzyl (a semisynthetic derivative of glycyrrhizinic acid) displayed cytotoxicity in MIAPaCa-2 (IC_50_: 7 µM) [115]. Various studies have shown licorice’s inhibitory and protective properties and derivatives against carcinogen-induced DNA damage [116]. At last, it is concluded that licorice and its derivatives should be reviewed, and rationale will be suggested for the combinations of agents via clinical trials.

#### 5.1.6. Effect on Prostate Cancer

**Figure 3:**
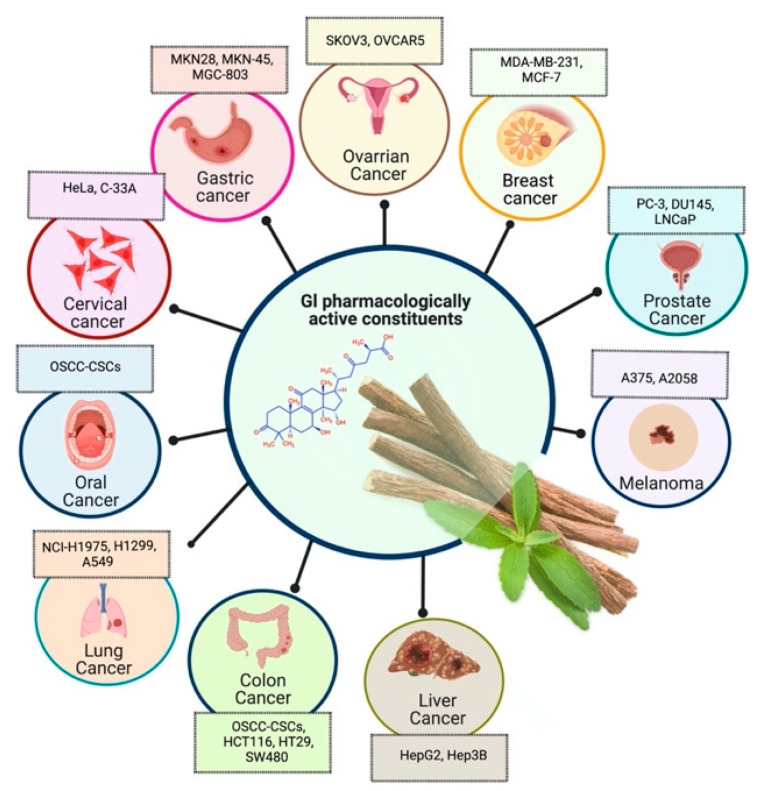
Pharmacological efficacy of *G. glabra* against different cancer and their respective cell lines.

Prostate cancer is the most common noncutaneous cancer among men. Radiation therapy, androgen deprivation therapy, and combination chemotherapy are the most common conventional therapies for prostate cancer [111,117]. In recent years, herbal therapies have been commonly used in western countries. According to a recent study, prostate cancer cells LNCaP and C4-2 were significantly inhibited by ISL in a dose-dependent manner. In addition, it lessens the mitochondrial membrane potential [Psi(m)] and level of ROS while there was no aftermath on intraepithelial carcinoma-6 epithelial cells. C4-2 cells were selectively inhibited by abnormal AMP-reliant/stimulated ERK and protein kinase pathways [118]. Isoliquiritigenin inhibited cell cycle progression in DU145 human and MatLyLu rat prostate cancer cells, resulting in antitumorigenic effects. Isoliquiritigenin boosted cell cycle capture by lessening cyclin E, cyclin D1, and cyclin-reliant kinase-4 protein levels and increasing the magnitude of cells in the G1 phase [119]. The pharmacological efficacy of *G. glabra* against different cancer and their respective cell lines has been shown in Figure 3.

### 5.2. Licorice in the Treatment of Respiratory Tract Infections

Breathing difficulties accountable for chronic obstructive pulmonary disease (COPD) is a type of lung condition. Chronic bronchitis and emphysema are examples of it. When air sacs are damaged, this lung disease is known as emphysema, and long-term inflammation in the airways is chronic bronchitis. Asthma, COPD, and acute respiratory distress syndrome (ARDS) are caused due to airway inflammation. Anti-inflammatory treatments effectively treat respiratory tract infections were validated by various studies. However, the worldwide leading cause of high mortality is COPD, and a significant factor is cigarette smoke. Chronic inflammation and oxidative stress are causes of COPD, which is due to lung dysfunctions. For thousands of years, herbal drugs have been used to cure numerous illnesses; they exhibit promising results and enhance physical performance [120,121,122]. This section has reviewed the *Glycyrrhiza glabra* literature as the potential therapeutic compound to cure pulmonary inflammation.

**Figure 4:**
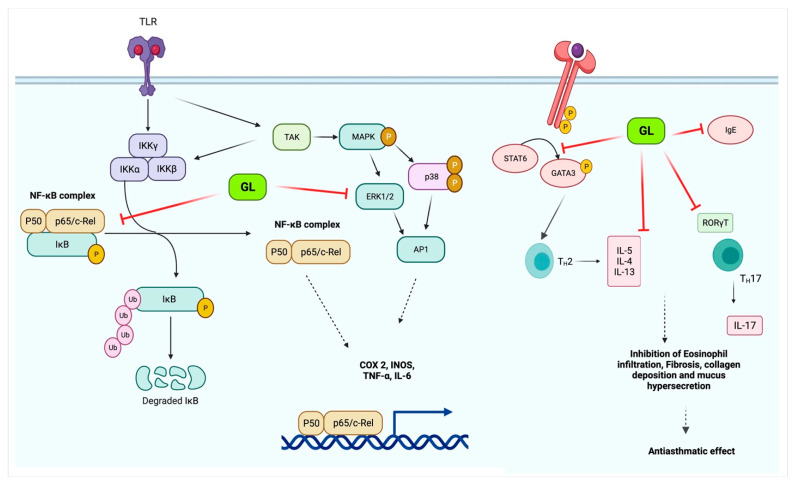
*Glycyrrhiza glabra’s* probable anti-asthmatic mechanism of action.

Isoliquiritigenin is a natural flavonoid that is derived from the root of the licorice. Isoliquiritigenin has exhibited anti-inflammatory and antioxidant properties. Researchers had tested the effect of isoliquiritigenin in a mice study on cigarette smoke-induced COPD. This study’s outcomes have demonstrated that isoliquiritigenin has lessened inflammatory cells’ infiltration and inflammatory cytokines. In addition, isoliquiritigenin regulated the NF-кβ and Nrf2 signaling pathways and protected against cigarette smoke-induced COPD [123]. In another study, a mouse model was used to know the efficacy of the herbal medicinal combination of *Agastache rugosa*, *Glycyrrhiza glabra* containing glycyrrhizic acid, the active constituents to treat COPD. It has shown this combination effective as an anti-COPD agent. This combination is more effective than alone *Glycyrrhiza glabra* or *Agastache rugosa* alone also reduce histopathological lung injury. Furthermore, glycyrrhizic acid and flavonoids, the *Glycyrrhiza glabra*’s significant components, have shown anti-asthmatic effects [124]. *Glycyrrhiza glabra’s* probable anti-asthmatic mechanism of action is shown in Figure 4.

A clinical trial was conducted to conclude in *Boswellia carterii* (Olibanum) and *Glycyrrhiza glabra* as broncho relaxants. Chronic bronchial asthma affected 54 patients who participated in this trial. Clinical examinations have been conducted such as serum electrolytes test: calcium, selenium, calcium, and potassium with pulmonary functions tests. *Glycyrrhiza glabra* has shown superiority over *Boswellia carterii* to manage chronic bronchial asthma [125]. Glycyrrhizin assists in the inhibition of fibrosarcomas and lung cancer [126]. Glycyrrhetinic acid has exhibited inhibition of bile acid-induced necrosis and apoptosis [127,128].

**Figure 5:**
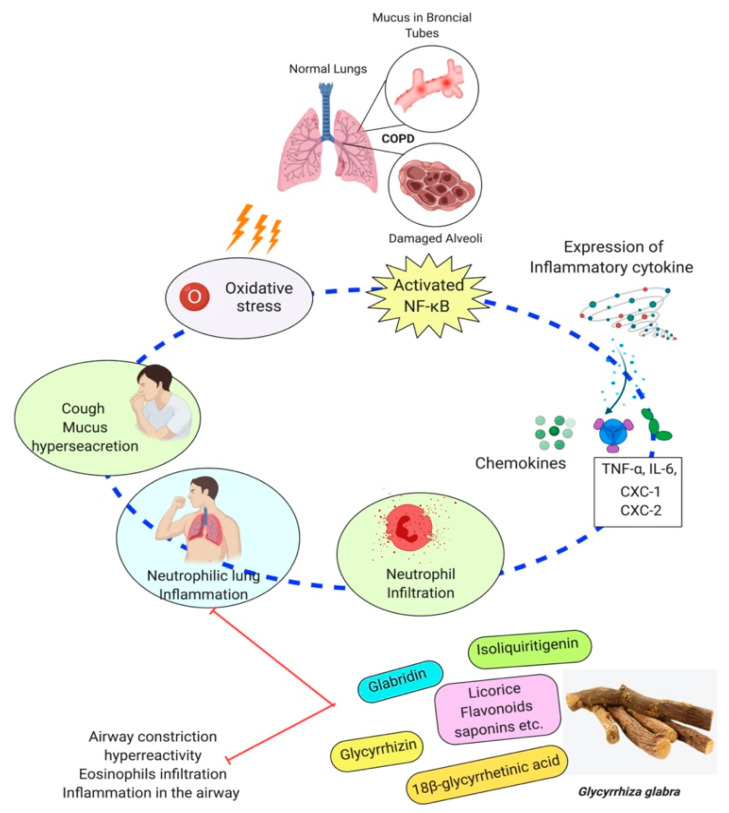
Molecular events involved in COPD’s pathogenesis and their possible modulation by *Glycyrrhiza glabra.*

The 18β-glycyrrhetinic acid and glycyrrhizic acid were reported to lessen the inflammatory cytokines generation. According to this study, both 18β-glycyrrhetinic acid and glycyrrhizic acid could be important biological inhibitors for remedying lung inflammation [129]. Glycyrrhizic acid was used in a mice model to treat irradiation-induced pneumonitis/fibrosis. The results conclude that glycyrrhizic acid might be the potential agent as an anti-irradiation lung injury drug [130]. The outcome of a study reported that the licorice flavonoids successfully diminish LPD-stimulated pulmonary inflammation due to suppression of cells intrusion and inflammatory intermediary, which come from lessening in neutrophil enrollment into lung neutrophil-intermediated oxidative injury. After effect of the study concluded that flavonoid extracts of licorice are anti-inflammatory compounds [131]. *Glycyrrhiza glabra has shown its effectiveness through many studies in inhibiting* airway constriction, hyperreactivity, eosinophils infiltration, remodeling, and inflammation in the airway [132,133]. Molecular events involved in COPD’s pathogenesis and their possible modulation by *Glycyrrhiza glabra* are shown in Figure 5.

An inflammatory mice model was used to examine the effect of glabridin ovalbumin stimulated airway hyperresponsiveness. It was found that glabridin may have the potential to treat asthma. Glabridin anti-inflammatory action is mediated by reducing the level of serum IgE, total protein, WBC count and improve respiratory function [134]. The preclinical model’s findings show that quercetin has anti-inflammatory and antioxidant properties reduced to inflammation, oxidative stress with neutralising free radical species, enhancing antioxidant enzymes’ expression. Furthermore, quercetin competes for adenosine triphosphate (ATP) binding sites to inhibit various protein, and lipid kinases reduce inflammatory pathways [135]. In addition, there are saponins in licorice root that help loosen the build-up mucus to be expelled more easily from the lungs. Unfortunately, respiratory tract infections cause mortality and morbidity, and the current standard therapies are not adequate. We can explore licorice as a potential remedy for obstructive respiratory diseases and COPD using the literature, animal models, and human trials.

### 5.3. Licorice Effect on Cardiovascular System

Three hundred active components are found in licorice, used for thousands of years. The principal functioning component of licorice is glycyrrhizin. Glycyrrhizin is a prodrug of licorice transformed 3β-monoglucuronyl-18β glycyrrhetinic acid (3MGA) and 18β-glycyrrhetinic acids in the intestines. 3MGA and GA suppress the enzyme 11β-hydrogenase type II (11β-HSD2) that changes cortisol to cortisone. High cortisol levels result from a modest mineralocorticoid abundance in the kidney and boost systemic vascular resistance by provoking mineralocorticoid receptors. Continuous suppression of 11 beta-HSD2 due to excessive licorice consumption results in hypernatremia, hypokalemia, and high fluid content, leading to significant life-threatening consequences, particularly in individuals with cardiovascular disease. Meta-analyses with 26 and 18 investigations have reported that licorice consumption and blood pressure significantly increase systolic and diastolic. This study has shown that licorice consumption affects the human body and demonstrates the distinction between licorice’s health advantages and its potential for adverse effects [136]. Several pieces of research have been published on the impact of the various chemicals present in licorice root. Glabridin is a powerful antioxidant with hypoglycemic properties [137].

**Figure 6:**
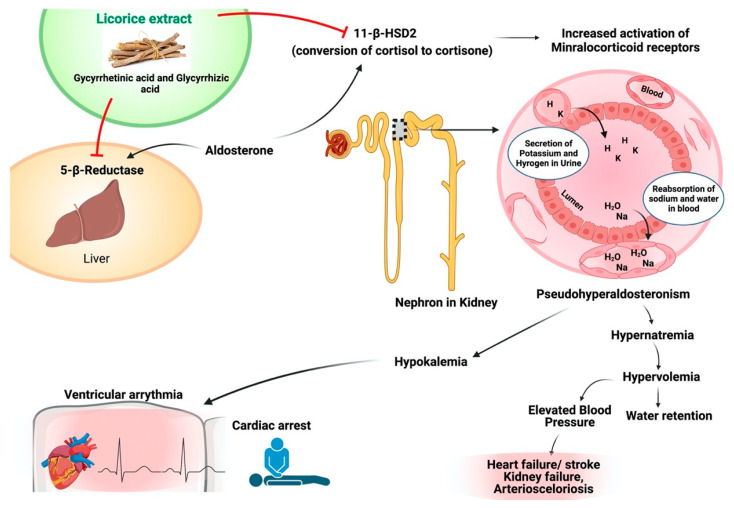
Possible mode of action of licorice against cardiovascular disease.

High licorice consumption may produce significant serious problems, people who already have increased blood pressure or take anti-hypertensive medications. Glycyrrhizin, 3MGA, and GA have been blamed for the negative consequences of high-dose licorice consumption. Therapeutic dosages of licorice have been considered safe in humans since the final toxicology assessment was released in 2007 [136,138]. Scandinavian nations have higher consumptions of licorice with higher content of GA [138]. This implies that the detrimental effects of licorice on cardiovascular health should be brought to the public’s attention. To minimize drug-induced mishaps, it’s critical to understand how licorice interacts with prescription medications. The possible mode of action of licorice against cardiovascular disease has been shown in Figure 6.

### 5.4. Licorice Effect on Hepatoprotective System

The liver conducts several critical processes (metabolism, detoxification, and bile production). It protects against foreign chemical exposure by detoxifying and removing them. Because the liver is responsible for the metabolism and elimination of medicines from the body, a healthy liver is critical to general health [139]. The liver might be damaged by excessive exposure to chemotherapeutic drugs, environmental pollutants, alcohol, drug overdose, carbon tetrachloride (CCl4), and thioacetamide, which could be a cause of cirrhosis, hepatitis, hepatitis, and alcoholic liver disease. Glycyrrhizin is a compound found in the licorice root. Various studies have shown that it has hepatoprotective properties to treat viral hepatitis, which could be cytoprotective action through TNF-α stimulate cytotoxicity inhibition and immune-intermediated cytotoxicity suppression opposing hepatocytes [140,141,142]. In TCM, licorice is widely used to treat liver disease [143]. It’s also utilized to decrease toxicity, increase appetite, and boost the efficacy of other prescription medicines [66]. Chronic hepatitis is caused by toxin exposure, viral infections, ischemic-reperfusion damage can all be treated with glycyrrhizin [144]. Magnesium salt (Magnesium isoglycyrrhizinate (MgIG)) is one of the stereoisomer 18- α of glycyrrhizic acid, a new molecule derived from licorice root [145]. Japan and China are using hepatoprotective medications to improve functions of the liver; these medications stabilize the cell membranes with the inhibition of liver inflammation [146]. MgIG treats inflammatory liver disease as an anti-inflammatory and hepatoprotective medication [147].

**Figure 7:**
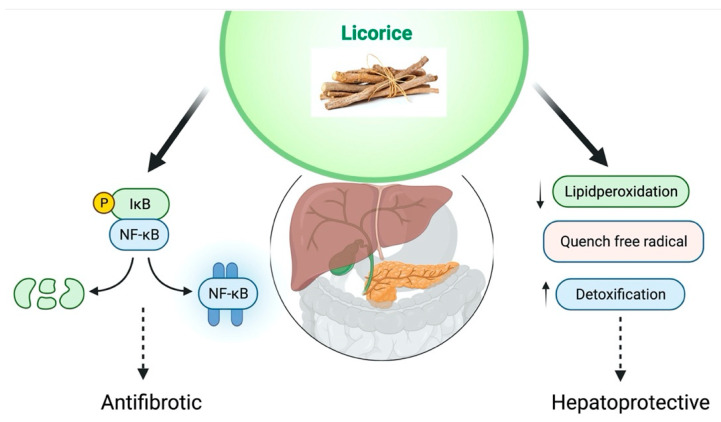
Proposed hepatoprotective effect of *G. glabra.*

Licorice flavonoid oil, glycyrrhizin, GA, and specific licorice preparations have potent hepatoprotective activities. Japan and China have developed glycyrrhizin as a hepatoprotective medication. GA has been shown the properties of hepatoprotective effect. The metabolic process in the human body converts GA into glycyrrhetinic acid; thus, glycyrrhetinic acid and GA have the same pharmacological properties [148]. GA has shown anti-inflammatory and antiapoptotic properties through the inhibition of TNF-𝛼 and caspase-3 that explains the hepatoprotective effect of GA. Liver regeneration could be aided by the expression of proliferating cell nuclear antigen that GA increases. Glycyrrhizin might be a potent medication protecting the liver from endotoxin-induced damage, particularly after a large hepatectomy [149]. Ischemia-reperfusion (I/R)-induced liver damage and the formation of high-mobility group box 1 (HMGB1) are both linked to activated Kupffer cells. GA, I/R-induced liver damage was avoided by inhibiting HMGB1 synthesis by Kupffer cells [150]. In vivo study in rats has shown that GA binds to lead; it is an efficient chemopreventive drug that leads to acetate-induced hepatic oxidative stress. GA inhibited CD4+ Tcell proliferation in response to ConA via the Jun N-terminal kinase (JNK), extracellular signal-regulated kinase (ERK), and phosphoinositide 3-kinase (PI3K)/AKT pathways, alleviating ConA-induced inflammation and fibrosis development in livers [151]. GA blocked the complement system’s lytic pathway, perhaps preventing tissue damage produced by the membrane assault complex. As a result, GA may be an effective inhibitor of complement-dependent tissue damage in autoimmune and inflammatory disorders [152]. It can be concluded that GA may help liver disease after reviewing these studies. The proposed hepatoprotective effect of *G. glabra* has been shown in Figure 7.

### 5.5. Antimicrobial Activity

Microorganisms build side effects and resistance against antibiotics. Therefore, biologically active compounds isolated from plant species and extracts have been got much attention to overcome. Medicinal plants provide a natural source in place of antibacterial agents. The antimicrobial activeness of the herb extracts and oils has been identified for several years and recorded that it may be associated with saponins, alkaloids, flavonoids, glycosides, phenols, and tannin [153,154].

Previous studies have described the antimicrobial activities of roots and rhizomes, but only a few reports have shown the effect of licorice leaves against microorganisms [155]. Ethanolic and aqueous extracts of the leaves of licorice were examined to evaluate the antimicrobial potency. Serial dilution method and paper disc evaluation method was applied to measure the minimum inhibitory concentration (MIC) and minimum bacterial concentration (MBC) to test the antimicrobial activeness of *Klebsiella pneumoniae*, *Candida albicans*, *Escherichia coli*, *Pseudomonas aeruginosa,* and *Enterococcus faecalis*. The results have confirmed that licorice’ ethanolic extract has antimicrobial potential against *Candida albicans* and gram-positive bacterial depending upon dose. Licorice’s ethanolic extract of leaves is potent against gram-positive bacteria; therefore, it can be the probable alternative medication against diverse strains [156].

Karahan F et al. investigated the antioxidant and antimicrobial properties of methanolic root extracts of *Glycyrrhiza glabra* var. *glandulifera*. Samples of the plants were collected from Turkey’s east Mediterranean part. MIC and disc-diffusion methods were employed to examine the antimicrobial effectiveness. The antimicrobial assays concluded that methanolic roots extracts were less effective against the Gram-negative bacteria than the Gram-positive bacteria. Furthermore, root methanolic extracts have shown more effective against *Candida* species than other bacteria. Results of the study have shown that environmental factors affect the content of chemical constituents and biological properties for the usual licorice in each habitat. In addition outcome of the study backed the traditional practices of licorice and advocated that it could be valuable to treat other infections [157]. Gupta VK et al. researched to examine the antimicrobial effect of *Glycyrrhiza glabra* roots, and they found the antimicrobial potential at the concentration of 500 µg/mL. Phytochemical analysis has exhibited that glabridin potentially inhibited H (37) Rv strains and *Mycobacterium tuberculosis* (H37) Ra at concentration 29.16 µg/mL. Thus, it has the antimicrobial potential to inhibit both Gram-positive and Gram-negative bacteria [158].

Antibacterial properties of *Glycyrrhiza glabra* were examined against *Bacillus ceeruis*, *Escherichia coli*, *Pseudomonas aeuruginosa,* and *staphylococcus aureus*. It was investigated by using agar well diffusion and dilution test methods. The outcomes of this study show that the highest effect was on *S. aureus* and the lowest impact on *P. aeruginosa*. Thus, the results of this study approve that *G. glabra* extract can be a potential treatment against bacterial infections. The study results established that *G. glabra* could be an alternative medication against bacterial agents [159]. Various studies concerning the antibacterial effect of licorice have been summarized in Table 2.

### 5.6. Anti-Inflammatory Activity

Inflammatory illnesses are becoming more common and have a more significant impact on daily life, prompting researchers to look for novel pharmaceutical ways to combat them. Currently rising use of nonsteroidal anti-inflammatory drugs to cure various discomfort and inflammation, but these drugs have several adverse effects. People’s interest in herbal medicine for the treatment of inflammation is also jumping up because herbal medicines have few or no adverse effects [19]. Medicinal plants and their constituents have taken part in the development of numerous drugs to medicate several ailments. *G. glabra* is one of the medicinal plants employed to medicate inflammatory diseases since ancient times [164,165]. This section article sums up the information on licorice and isolated compound it and their mechanism of action and establishes the new pave for the latest research to cure inflammatory diseases.

Licorice has shown anti-inflammatory activities due to decreasing PGE2, MMPs, TNF, and free radicals validated by its traditional uses such as relieving coughing, eliminating phlegm, stimulating digestive functions, alleviating pain, and many others more [164]. In CIA rats, licorice processed DGN products dramatically reduced RA symptoms. Matrix metalloproteinases, inflammatory cytokines, and vascular endothelial growth factors were all regulated by licorice processed DGN products in blood and cell supernatants. This study concluded that licorice-processed DGN products have shown anti-inflammatory effects through TLR4/NF-кβ/NLRP3 signaling pathway on CIA rats and LPS-induced RAW264.7 cells and regulated the metabolic profile in managing RA [166]. In vivo anti-inflammatory activities have been shown by total flavonoids isolated from licorice extracts and licorice via suppressing COX-2 gene, iNOS, and signals of mitogen-activated protein kinases (MAPKs) [167,168]. Flavonoids are keeping multiple pathway integrated mechanism of action, therefore, showing anti-inflammatory properties. As a result, flavonoids of licorice are the potential medication for inflammation with minor adverse effects [169].

Wang et al. carried research to examine the consequence of glycyrrhizin in mice for anti-inflammatory treatment and investigate the possible actions of mechanism. The study results have exhibited that expression levels of iNOS, COX-2, TNF-α, and IL-6 were significantly decreased by the glycyrrhizin, a triterpene of licorice. The study results found that glycyrrhizin acts as an analgesic by attenuating the expression levels of COX-2, TNF-α, iNOS, and IL-6 [24]. It significantly attenuated the expression of iNOS and IL-1𝛽 and decreased the levels of MDA and NO at the site of inflammation [170]. A study was conducted to examine the protective effect of isoliquiritigenin, a flavonoid monomer. Isoliquiritigenin lessens oxidative stress by modulating the Nrf2/HO-1, reducing acute pancreatitis in a pancreatitis model [171,172]. Secondary metabolites and licorice extracts have shown anti-inflammatory activities to treat various diseases in addition to acute kidney disease. Isoliquiritigenin reduces LPS-stimulated acute kidney damage by the suppression of NF-кβ and TNF-α stimulated formation HMGB [173,174]. Isoliquiritigenin reduces the inflammation and fibrosis in the kidneys caused by unilateral ureteral inhibition [175]. Isoliquiritigenin also inhibited inflammatory cytokines, excessive deposits, and the NF-кβ and Nrf2 pathways, all involved in the Ang II-stimulated hypertensive renal damage [176]. Neutrophils produce ROS at the inflammation site, which causes lessened tissue injury by licorice and glycyrrhizin extract [177]. H5NI induces ROS, inhibiting it by glycyrrhizin via suppressing JNK, NF-кβ, p38, and inhibiting H5N1replication in the lung cells. H5N1 also stimulated pro-inflammatory gene expression [178]. Plasma immunoglobulin E (IgE) and ovalbumin stimulated bronchial asthma models were used to investigate the outcome of three different doses of licorice extract on the bronchoalveolar lavage oxidative stress indicators. It decreased the level of interleukin IL-13, (IL)-5, and IgE. The study results have shown that licorice 10 mg/kg inhibits the mucus and protects against OVA-induced lung inflammation. It is concluded in this study that the lowest dose of licorice is more effective against anti-inflammatory and antioxidant action [179]. Licochalcone-A showed anti-inflammatory action by inhibiting MMP1, MMP3, and MMP13 production in IL-1β stimulated chondrocytes [180]. While licochalcone-C anti-inflammatory action mediated by decreasing NF-кβ, and other downstream molecules, such as inducible iNOS, ICAM-1, VCAM-1 [181].

### 5.7. Dental Caries

Dental caries is public health issue since it is one of the most frequent illnesses worldwide. Global Burden of Disease in 2017 reported that the most prevalent health problem is untreated dental caries (tooth decay) in permanent teeth. In the last 30 years, low-income and vulnerable people have been more impacted by dental caries than high socioeconomic groups. The plaque formation on the outside teeth transforms the sugar in beverages and meals into acids that eventually destroy the tooth. Lack of plaque clearance by toothbrushing, high free sugar intake, and inadequate fluoride exposure cause discomfort, caries, infection, and occasionally tooth loss [182,183,184]. Aciduric/acidogenic bacteria incursion in dental plaque that is also recognized as dental biofilm causes the root cause of gradual deterioration of the complex tooth structure; this complication is known as dental caries. Primary etiological agents of dental caries are mutants streptococci (*Streptococcus sobrinus* and *Streptococcus mutans*), and other contributors are *Actinomyces* spp. and *Lactobacillus* spp. of tooth decay [185,186]. It is a common chronic infectious, transmissible illness caused by tooth-adherent bacteria, principally *Streptococcus Mutans*, which metabolize carbohydrates to create acid, demineralizing the tooth structure over time [187].

One of the most prevalent health problems in children is dental caries. 60 percent of children in the middle ages of 5 and 17 years have deteriorated, rotten or lost, or misplaced permanent teeth problems in the US. In a pre-school setting, a pilot study for young children was conducted to investigate the consequence of using the protocol of herbal caries-prevention to suppress the *Streptococcus mutants*. Licorice root extract containing sugar-free lollipops were formed and were administered three weeks twice daily to children. SM counts were determined by analyzing saliva for specific monoclonal antibodies. Three groups were formed low, medium, and high-risk employing SM extent as to risk index. Bacterial counts were compared during the treatment and after nine weeks of treatment. The trial results revealed that administering herbal lollipops two times a day lessened bacterial count and relative percent in high-risk children [188]. In addition, there were studies conducted to establish the anti-cariogenic properties of licorice. *G. glabra’s* primary, secondary metabolites, glycoside, and triterpenoid saponin have been the topic of various investigations [189]. According to a study, Glycyrrhizin suppresses the glucosyltransferase property of *S. mutants*, that concern the formation of insoluble glucans essential for biofilm development. Acidulated phosphate-fluoride solution of glycyrrhizin lessens solubility due to surface coating effects and settlement in the porous structure of enamel demineralization and enhance fluoride absorption [190]. Glycyrrhizin has shown no significant impact concerning mineral loss in artificial caries lesions, validated by in vivo study. This might be due to a lack of glycyrrhizin concentration or exposure duration [191]. In addition, glycyrrhizin lessens enamel disintegration by suppressing acid production in dental plaque [192]. These studies pave the way for randomized clinical studies of licorice in the different administration forms as herbal lollipops.

### 5.8. Other Pharmacological Effects

Cancerous conditions that affect the mouth are called oral submucous fibrosis (OSF). Existing therapies only give short symptomatic alleviation at this time, and there is no viable treatment for OSF. Therefore, a study was designed to examine the inhibitory effect of isoflavane and glabridin extracted from licorice root. This study investigated the fibrotic buccal mucosal fibroblasts in humans on features of the myofibroblast. The outcomes of the study have displayed that myofibroblast activities were inhibited by glabridin in a dose-dependent manner. Glabridin also inhibited arecoline-induced myofibroblast activation; therefore, it could be employed as a natural anti-fibrosis medication to treat the OSF [193]. Glabridin is a hydrophobic antimelanogenic substance derived from licorice root extracts. Using a human skin model, the effects of cationic glabridin-containing polymeric micelles produced from PMCP (Glabridin/PMCP-PM) were evaluated on glabridin’s capacity to enter the skin and suppress melanogenesis. Glabridin/PMCP-PM has shown promise as a transdermal delivery method for treating skin hyperpigmentation. Therefore, glabridin is extensively used in cosmetics because of its ability to enhance pigmentation [194].

A study has shown that licorice extract was more effective on tyrosinase activity than the extract of glabridin content with predicted based. As a result, we looked for other components that may be involved in the significant inhibitory action. According to the findings, the licorice extract’s glabrene and isoliquiritigenin block mono-and diphenolase tyrosinase activities. Isoflavones and chalcones have been presented as possibilities for skin-lightening agents [195]. Natural medicine options are deficient in curing acute ischemic stroke. Several neuroprotective chemicals have been discovered in licorice root. 75 patients with acute ischemic stroke were admitted to the neurology emergency department at Namazi hospital, connected with Shiraz University of Medical Sciences in Iran. 75 sufferers of acute ischemic stroke were given 450 or 900 mg licorice extract or placebo capsule doses three times for seven days. The trial’s outcomes were impressive, and proposed using whole licorice extract to help individuals with acute ischemic stroke alleviate their neurologic symptoms. Licorice could be beneficial as a therapy for sufferers with symptoms and acute ischemic stroke [196]. In vitro studies have shown that neuroprotective medications such as isoliquiritin and liquiritin effectively inhibit glutamate-mediated cytotoxicity following hypoxic injury to brain tissue. [197,198].

Conjugated active molecules glycyrrhetinic acid, and glucuronic acid forms the glycyrrhizic acid that is a triterpene. All three medicines are metabolically active and have shown antioxidant, anti-inflammatory, and antiviral properties [199]. In live mice, flavonoids have been shown to exhibit neuroprotective properties [197,198,200]. Three triterpenoids and five flavonoids were extracted from licorice extracts, and they have demonstrated potential antidiabetic activities in vitro and in vivo. This was accomplished through various mechanisms of action such as enhancing sensitivity and appetency of insulin receptor sites to insulin, improving glucose utilization in multiple tissues and organs, resisting peroxidation, clearing free radicals, enhancing microcirculation, and correcting lipid and protein metabolic disorders in the body. Multiple signaling pathways, including the AMPK, PI3K/Akt, MAPK, AGE-RAGE, NLRP3, and NF-кβ, signaling pathways, target the licorice compounds [201]. Rich flavones are found in the ethanolic extract of *G. glabra*. Glabridin, glabrol, glabrene, 4′-O-methylglabridin, and four hydrophobic flavonoids are the main constituents of *G. glabra*, and they are employed to cure diabetic nephropathy and chronic hyperglycemia and loss of skeletal muscle [202,203,204,205]. Licorice and its metabolites offer a lot of medicinal promise for diabetic mellitus therapy. *In vivo*, in vitro, and human trials have shown that licorice and secondary metabolites have potential antidiabetic activities. Thus, licorice has the potential to be used as a natural therapy for a variety of ailments. However, to assess its pharmaceutical potential, more profound knowledge of its pharmacological processes is required.

## 6. Clinical Studies

Bardhan et al. conducted a clinical study to investigate the outcome of licorice on 96 patients with gastric ulcers, treated them randomly with placebo or deglycyrrhizinated licorice. However, no difference was observed after four weeks of treatment in the patient’s percentage ulcer area or clinical conditions [206]. A clinical study was carried on 66 patients to determine the impact of licorice on non-alcoholic fatty liver disease (NAFLD). They were divided into two groups, case and control, and it was a randomized, double-blind clinical trial. The patients were split into two groups: those given licorice and those given a placebo. The mean AST and ALT levels in the licorice group fell considerably from 58.18 to 49.45 (U/L) and 64.09 to 51.27 (U/L), respectively (all *p* < 0.001). There was no statistical effect in the control group. There was no BMI difference after and before the trial, and it was not significant statistically. The study’s outcome validated a substantial decrease in liver enzymes by the intake of licorice root extract [207].

Many mechanisms might explain the cause of extended liver enzyme levels. Glycyrrhizin’s anti-inflammatory action in the liver may be linked to enhanced hepatic lymphocyte activity, decreased production of IL-10 by liver dendritic cells, and inhibition of the complement’s lytic pathway [152,208,209,210]. The goal of this trial was to see how effective and safe glycyrrhizin was in treating severe acute exacerbations (SAE) of chronic hepatitis B (CHB). Randomly two groups of SAE and CHB were treated with tenofovir plus intravenous glycyrrhizin and tenofovir. The study outcome indicated that serum level ALT and AST significantly lessened from baseline at 3, 5, 8, and 15 days treated with tenofovir plus intravenous glycyrrhizin. Week 1 and week 2, there were no relative changes found to baseline who were treated with tenofovir. Thus, for individuals with SAE of CHB, early administration of glycyrrhizin can be both safe and beneficial [211]. Several human studies have investigated the effect of glycyrrhizin on chronic HCV infection; outcomes of these studies have exhibited that glycyrrhizin is adequate to protect and treat chronic hepatitis C patients [212]. A clinical trial examined the effect of glycyrrhizin on European patients with hepatitis C virus (HCV)-RNA, and serum ALT and safety were tested. Fifty-seven chronic hepatitis C patients were randomly assigned to one of four dosage groups: 80, 160, or 240 mg glycyrrhizin or placebo. 240 mg dose of glycyrrhizin thrice-weekly does not affect HCV-RNA levels and lowers the serum ALT during treatment, and it is well tolerated and safe [213]. Glycyrrhizin therapy slows the development of liver disorder to hepatocellular carcinoma in patients with chronic hepatitis C. Glycyrrhizin was executed I.V three or six times per week to examine the feasibility and efficacy of serum ALT. The mean percentage was found serum ALT level 26% and 47% for the three times and six times per week patients treatment groups. The study’s outcomes have shown that medication six times per week is more effective than three times per week [214].

Although the mechanism behind its antiviral action isn’t entirely known. Glycyrrhizin lowers serum ALT through hepatocyte membrane stability, according to one theory [215]. In another study, it is found that T cell cytotoxicity is suppressed and increased, as well as activation of endogenous IFNs production. IFN-c, IL-12, and Th1 cytokines are crucial for virus clearance and T cell-mediated immunity [216]. Retrospective research examined the effect of glycyrrhizin therapy on hepatocarcinogenesis in interferon (IFN)-resistant hepatitis C. According to the findings, long-term glycyrrhizin injectable treatment lowers the HCC incidence in patients with IFN-resistant HCV-related chronic hepatitis and cirrhosis [217]. However, there is a need for more valuable data as six times weekly mode of administration to validate this administration dose.

In recent years, antibiotic resistance with *H. pylori* has increased and reduced the effectiveness of the antibiotic treatment [218]. 120 individuals with fast urease tests were split into two groups: the control groups were administered a triple regimen of clarithromycin, and the research group was administered licorice. Six weeks following treatment, *H. pylori* eradication was evaluated. The study’s outcomes have shown that the combination therapy of licorice with triple administration of clarithromycin improves the eradication of *H. pylori* in the case of peptic ulcers [219]. Another study found that the licorice group had an 83.3 percent favorable therapeutic response than 62.5 percent in the control group (*p* = 0.018). By blocking the dihydrofolate reductase enzyme and inhibiting DNA gyrase, a key enzyme for bacterial transcription and replication, licorice has anti-adhesive and antibacterial properties against *H. pylori*. Polysaccharides and aqueous extracts derived from the roots of *Glycyrrhiza glabra* have shown an antiadhesive property that might be used to develop cytoprotective preparations with anti-infectious potential [220]. A clinical study was conducted to find out the effectiveness of glycyrrhizin on 21 dental students. Glycyrrhizin was applied using a split-mouth method. Participants were told to stop using all forms of dental hygiene, but no dietary changes were required. After comparing both the groups, there was a notable decline in plaque in upper central incisors after three days in the experimental group. This discrepancy indicated a statistically significant difference. After 4 days, experimental sides had shown less plaque between the two halves of the mouths than three days. This trial has validated that glycyrrhizin has the potential to inhibit tooth plaque [221]. Some important clinical studies and their outcome has been shown in Table 3.

## 7. *Glycyrrhiza glabra* Toxicological Effects

Given the widespread desire to use herbal medicine, it is critical to demonstrate the negative consequences on health. There are currently misconceptions about the safety of herbal medications. Licorice (*Glycyrrhiza glabra)* has been used as a natural medicine since ancient times. Licorice is a familiar household name in various parts of the world with valuable medicinal properties. However, some studies have shown that licorice has adverse health consequences. In this section of the article, we compiled scientific research literature on their adverse effects to emphasize the safety of glycyrrhizin and licorice. Licorice is an extensively used herbal medication, and it is critical to assess its safety systematically. Licorice is considered a safe, natural, and effective culinary medication, but it is essential to pay attention to the dosage and duration. Some studies have shown the toxicity of licorice depending upon the dosage and duration. The root of the *Glycyrrhiza glabra* plant is used to derive the licorice, which has a mineralocorticoid-like action and contains the herbal component glycyrrhizic acid. Chronic licorice consumption causes a condition comparable to primary hyperaldosteronism. Excessive licorice consumption can lead to hyper mineralocorticoidism, a condition marked by salt retention, hypokalemia, hypertension, metabolic alkalosis, hypoaldosteronism, and low renin activity [222].

In addition, high amounts of glycyrrhizin can produce the effects of hypermineralocorticoids. Licorice and glycyrrhetic acid saponins may block the enzyme 11-β-hydroxysteroid dehydrogenase; due to it, cortisol promotes the mineralocorticoid action with the susceptibility of rising sodium and fall of potassium [223]. A lady of 34 years of age was suspicious of having experienced acute toxication after ingesting licorice for several months. LC-MS/MS was applied to assess the extent of licorice components as glycyrrhizin and glycyrrhetic acid with a mineralocorticoid action. A sensitive and quick method was used for quantifying glycyrrhetic acid, which included a simple sample preparation. 200 g of licorice had to be consumed in a licorice ingestion experiment. Because only amounts of glycyrrhetic acid were detected in the dead woman’s stomach and blood, the potential of acute fatal glycyrrhetic acid poisoning was ruled out [224]. Daily consumptions of licorice who have mineralocorticoid excess syndromes turn out to differ extensively (1.5–250 g/day), according to in vivo tests and clinical data [223]. *Glycyrrhiza glabra* may induce bloating and retention of water amid premenstrual syndrome [12]. According to a case study, excessive licorice consumption has been linked to edema, hypokalemia, and thrombocytopenia. It demonstrates that consuming too much licorice can have a toxic consequence in the form of thrombocytopenia [225]. A licorice candy cigar is made up of glycyrrhizic acid that is the constituent of licorice extract. A 49-year-old lady had weight gain, peripheral edema, and relative hypertension after consuming these licorice candies. Glycyrrhizic acid reaction subsided spontaneously for the patient. However, health workers should know the hazardous consequences of natural licorice extract to prevent symptom development when symptoms are detected early. When patients come with unexplained hypertension, hypokalemia, edema, rhabdomyolysis, or myoglobinuria, emergency doctors should inquire about using medications that may include natural licorice extract. Emergency doctors should ask about the licorice extract medications if the patient comes with myoglobinuria, hypertension, edema, and unexplained hypokalemia [226].

A patient was presented with acute rhabdomyolysis and a lack of myoadenylate deaminase (MADA) due to chronic licorice intoxication. The patient underwent a clinical and laboratory examination, as well as a morphologic analysis of skeletal muscle. Hypokalemia is the most common sign of licorice intoxication, explaining most reported clinical symptoms and morphological abnormalities. The lack of MADA might be due to the licorice glycosides’ direct harmful impact. Lack of MADA and chronic licorice intoxication have been linked to histochemical, clinical, morphological, and biochemical changes that could be reversed with licorice withdrawal and potassium supplementation [227]. A study has shown that glycyrrhizin and *Glycyrrhiza glabra* is moderately toxic. Amid pregnancy, careful use of glycyrrhizin and *Glycyrrhiza glabra* is advisable. Glycyrrhizin and *Glycyrrhiza glabra* have shown the cytotoxic effects on cancerous cells selectively. Both have exhibited secondary side effects such as hypertension and hypokalemia. Hypokalemia increases licorice’s side effects, reduces type 2 11-beta hydroxysteroid dehydrogenase activities, anorexia nervosa, female sex, and hypertension [228]. More licorice intake may cause hypertension, water retention, sodium, hypokalemia, and suppress renin-aldosterone [229].

**Figure 8:**
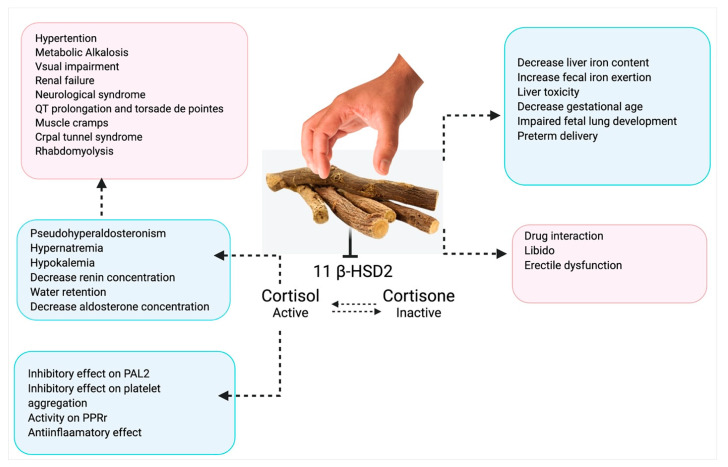
Toxicological effect of licorice.

Hereafter, the main challenge lies with the dosing of licorice, as licorice is available in different forms like beverages, candies, supplements, and extracts. All these have a varying amount of active constituents of licorice. The manufacturing of variable licorice dietary products is not strictly regulated in the United States of America also. The upper limit of intake glycyrrhizin recommended by the European Union in 1991 is 100 mg/day; this quantity is found approximately in 60–70 g licorice **[230]**. Clinical trials and in vivo studies have suggested a daily intake of glycyrrhizin 0.015–0.229 mg/kg body weight/day. Consumption of glycyrrhizin as licorice has shown low bioavailability, affecting humans and animals [223]. Male rats were given oral doses of 500, 1000, and 2000 mg/kg licorice extract; no substantial effect on reproductive function was detected. This study’s outcome displayed that prolonged licorice use may not produce any significant undesirable effect with the upper-limit dose of 2000 mg/kg [231]. Many reports have exhibited ocular complexities after the intake of licorice [232,233,234,235]. Gastrointestinal complications have been disclosed by the use of licorice and its medications, although most individuals recover without the need for additional treatment once the herbal medicine is stopped [236]. The reported toxicological effect of licorice has been shown in Figure 8.

## 8. Conclusions and Future Recommendation

Licorice is one of the most effective herbal medications for reducing toxicity and increasing the efficacy of other herbal medicines when used together. However, the biochemical research of licorice and its natural composition has been extensively studied, which still needs focus to authenticate its efficacy for healing various diseases. There is a requirement of studies on different compounds of licorice and their biological targets to know the mechanism of action. More studies are demanded to establish the synergy between the efficacy and toxicity of other constituents in combination preparations. In most cases, the advantages of licorice intake do not exceed the potential adverse effects. Many people in developing countries still depend on licorice extract to treat several human diseases. Researchers have developed various medicines with the aid of licorice. Comprehensive experiments and research have been carried out on the biological value of active constituents of this herb, and in vitro, in vivo, and human clinical studies provide us significant confirmation to take to the next level of research.

There is a need for a proper dosing schedule of licorice to cure different diseases; by this way, the use of licorice in pharmaceutical industries may increase its use, and it should be in a proper and controlled manner. This review concluded integrating isolated phytochemicals constituents from licorice and their biological role in fighting various physiological diseases and their secondary metabolites to develop promising pharmaceutical preparations. Thus, licorice can be helpful many conditions. To sum up the current review, licorice extracts and licorice flavonoids have been exploited for the activities such as hepatoprotective, anticancer, antibacterial, respiratory tract infections, and cardiovascular diseases. However, advance investigations are needed to extrapolate their mechanism of action in different biological activities. Furthermore, in-depth research and clinical trials are required on licorice to validate these pharmacological effects, to establish Glycyrrhiza plant extracts and its phytoconstituents as promising pharmaceutical and food ingredients and to fill some gaps in its safety and toxicological characteristics. In addition, well-designed research with various combinations of licorice formulations in various illnesses will remain an area of future research. We expect that these future studies will provide the base for the new advancements and usages of licorice.

## Figures and Tables

**Table 1:** Secondary Metabolites of *Glycyrrhiza glabra* and their mechanisms of actions.

Compound Name	Structure	Phytochemistry	Mechanism of Action	Reference
Glycyrrhizin	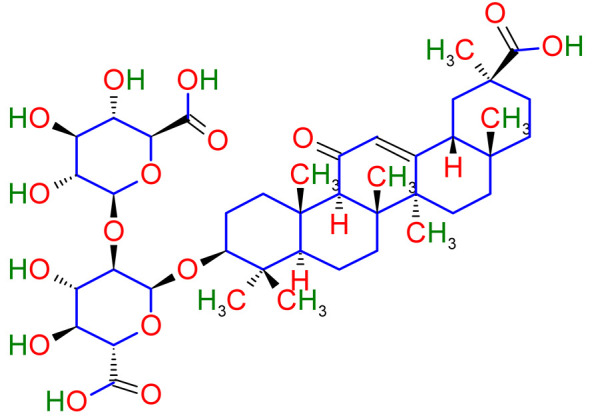	The main constituents are triterpene, saponins, and flavonoids.	Inhibited the prostaglandin, specifically prostaglandin E2 and cyclooxygenase activity as well as platelet aggregation.	[25,28,29]
Glycyrrhetinic acid	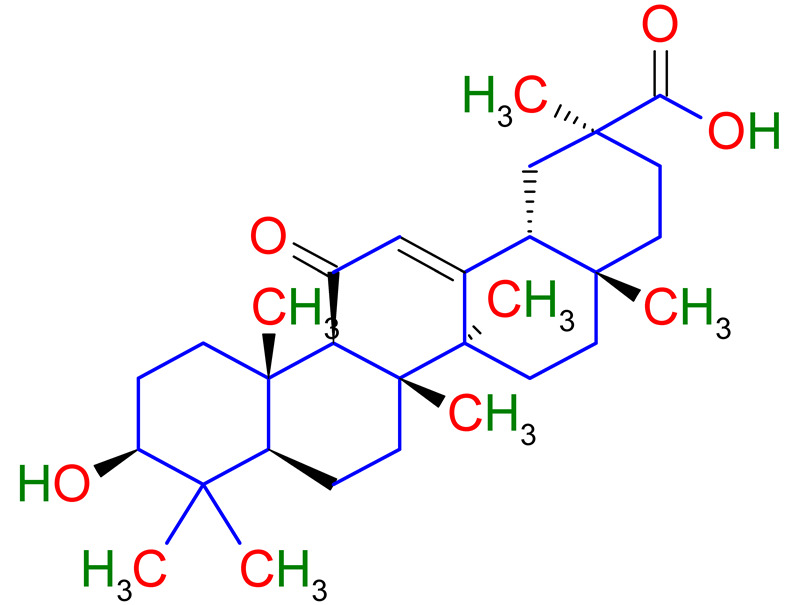	Active phytoconstituents are 18β-glycyrrhetinic acid, isoflavones, glabrin A and B, and glycyrrhizin.	Glycyrrhetinic acid has shown anti-inflammatory activity and inhibited 11β-hydroxysteroid dehydrogenase	[27]
Glabridin	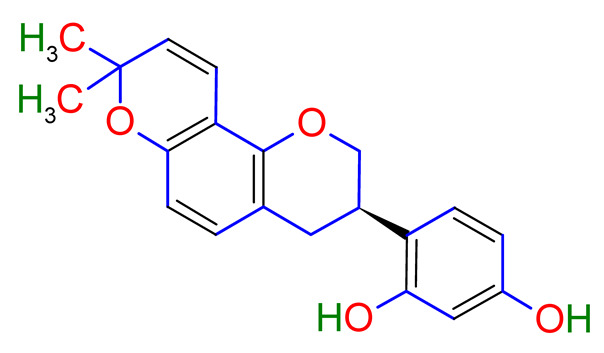	Glabridin is an isoflavane, a type of isoflavonoid. This product is part of a more prominent family of plant-derived molecules, the natural phenols.	Glabridin inhibited melanogenesis by two mechanisms (1) inhibited the production of ROS (2) inhibited tyrosine.	[25,28,30]
Quercetin	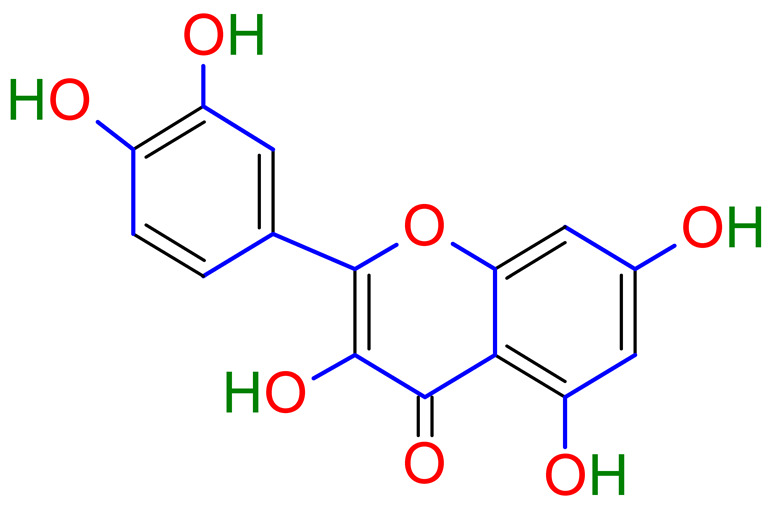	Plant-derived flavonoid.	How flavonoids inhibited enzymes is not known. It inhibits lipoxygenase and cyclooxygenase activities and decreases the production of inflammatory metabolites.	[31]
Liquiritigenin	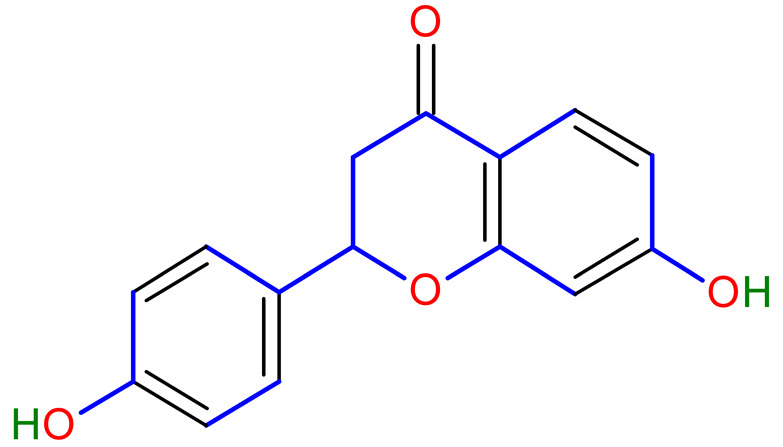	Phenolic compounds,	It is inhibited through the pathways NLRP3 and NF-кβ.	[32]
Isoliquiritigenin	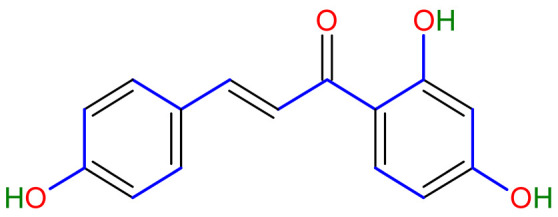	Phenolic compounds,	Reduce the inflammatory response of macrophages via the inhibition of the activation of AP-1, NF-кβ, and AP-1.	[33,34,35,36,37,38,39]
Licochalcone C	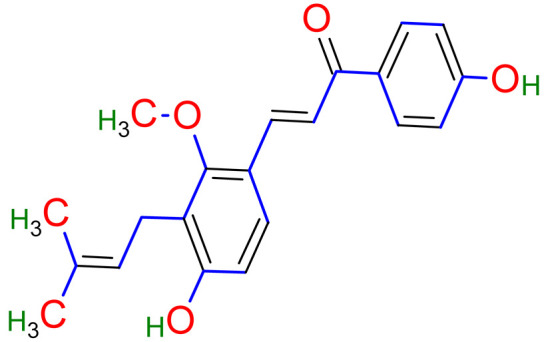	Phenolic compounds,	Electron transport in the bacterial respiratory chain is inhibited.	[40]
Formononetin	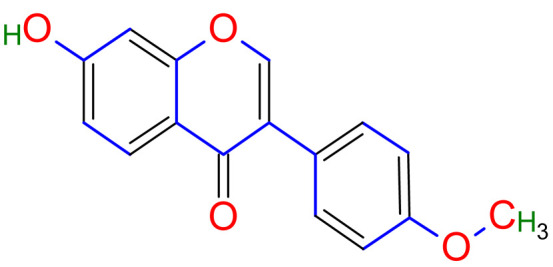	Bioactive isoflavones	They were arresting the cell cycle, inducing apoptosis, stopping metastasis via targeting numerous pathways.	[41]
Licopyranocoumarin	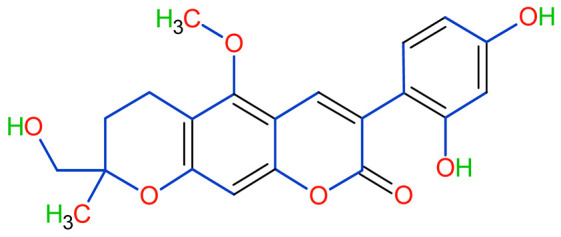	Coumarins	Without sny cytotoxicity, it inhibited the production of cells in HIV-infected cell cultures.	[42,43]
Glabrocoumarin	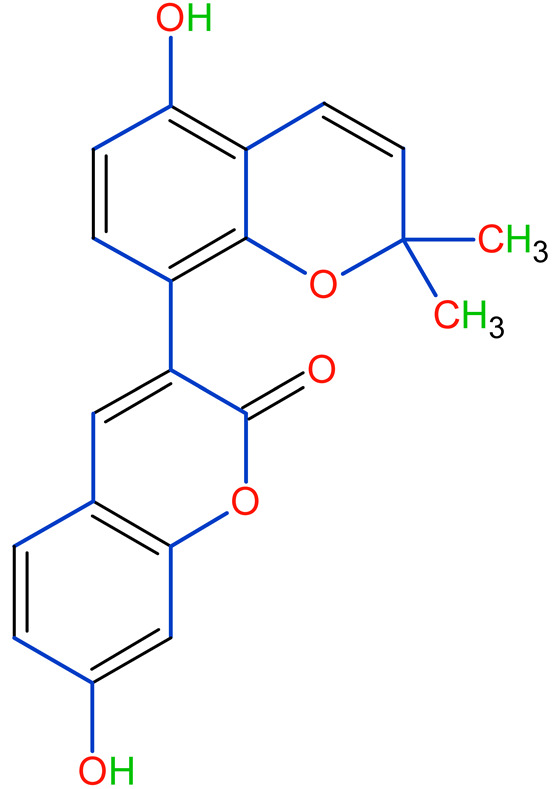	Coumarins	Without causing any cytotoxicity, it inhibited the formation of cells in HIV-infected cell cultures.	[42,43]
Kanzonol Y	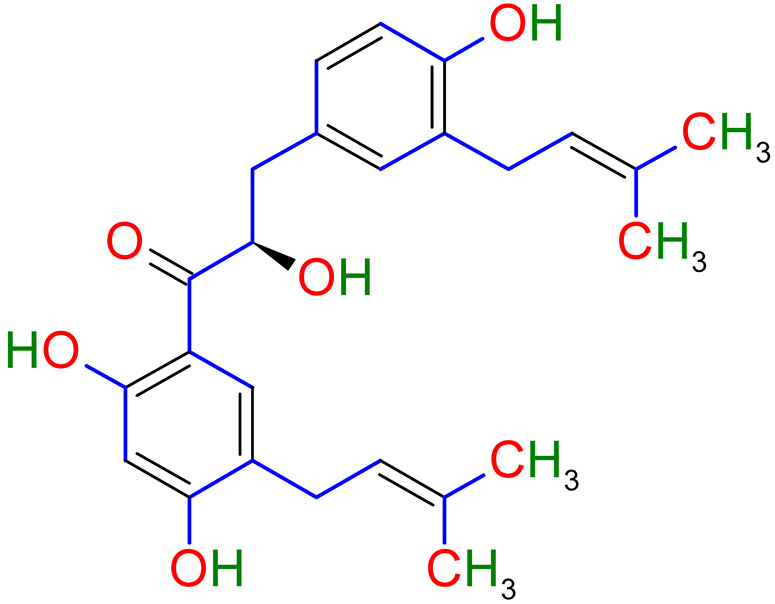	Chalcone	Inhibitory activity against Bacillus subtilis H17	[44]
Paratocarpin B	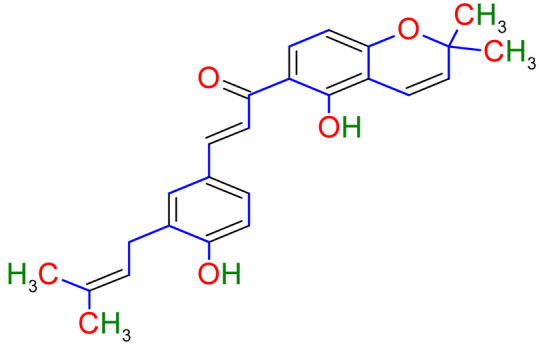	Chalcone	Peroxynitrite antioxidant assay has shown the antioxidant property. It is the most potent antioxidant agent.	[35,45,46]
Glycyglabrone	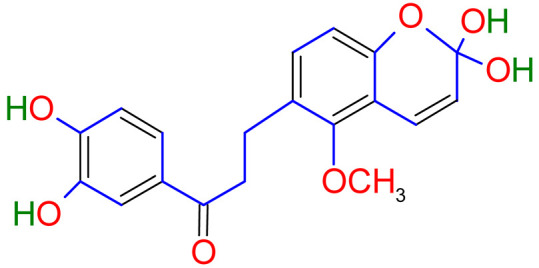	chalcone	It exhibited potent free radical scavenging activity.	[35,45,46]
Mannopyranosyl-D glucitol	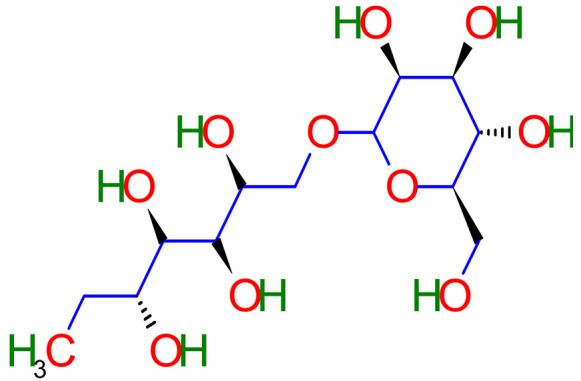	Mannose	Not reported	[47]
Glabridin	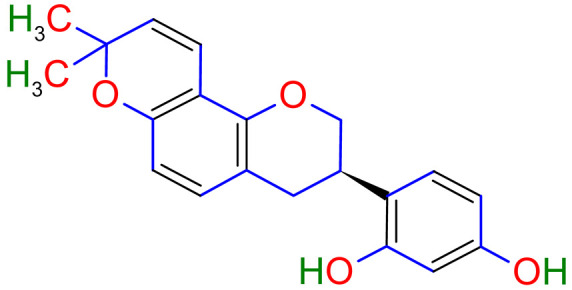	Isoflavones	Inhibitor of tyrosine.	[48]
Hispaglabridin B	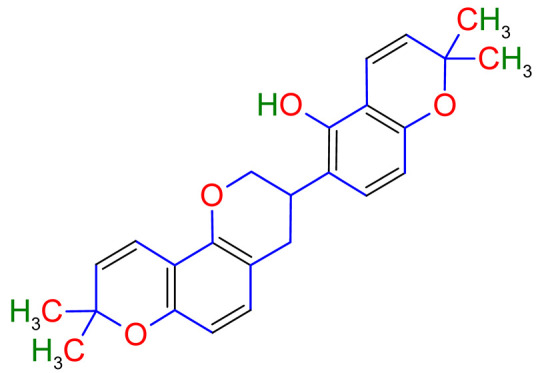	Isoflavones	It is the most potent antioxidant agent. FoxO1 transcriptional activity was inhibited via the expression of muscle-specific E3 ubiquitin ligases MuRF1 and Atrogin1 were decreased.	[35,49]
4-O-Methylglabridin	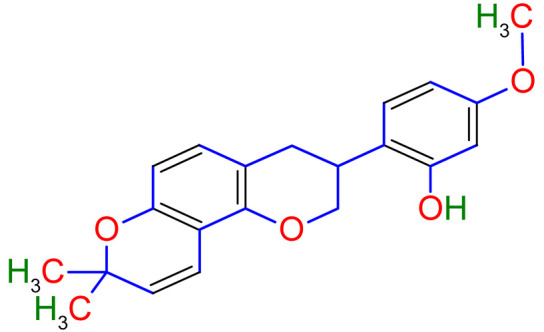	Isoflavans	Possess significant antimicrobial activity in vitro.	[50]

**Table 2:** Summary of studies showing the antibacterial effect of licorice.

Microbe	Methods	Antibacterial Effect	Extract Used	References
*Staphylococcus aureus, B. cereus, Pseudomonas aeruginosa*	Cell culture	Inhibited the growth	*G. glabra*	[157]
Oral pathogens	In vitro	Inhibited the growth of oral pathogens	*G. glabra*	[160]
*Mycobacterium tuberculosis* H(37)Ra and H(37) Rv strains	In vitro	Inhibited both Gram-positive and Gram-negative bacteria	*G. glabra*	[158]
*Staphylococcus aureus*, *Bacillus cereus*, *Pseudomonas aeruginosa* and *Escherichia coli*	In vitro	Inhibited growth of pathogens	*G. glabra*	[161]
*Salmonella typhi, S. paratyphi B, Shigella sonnei, S. flexneri,* and *enterotoxigenic E. coli.*	In vitro	Inhibited growth	*G. glabra*	[159]
*Candida albicans, Aspergillus niger, Aspergillus fumigates, Mucor* spp and *Penicilium marneffei*	In vitro	Inhibited growth of micro-organisms	*G. glabra*	[162]
*Staphylococcus aureus* and *Escherichia coli*	In vitro	Mild antibacterial effect	*G. glabra*	[163]

**Table 3:** Summary of clinical studies and their outcomes.

Participants	Interventions	Comparisons	Outcomes	Study Design	References/NCT Number
96 patients with gastric ulcers	Deglycyrrhizinated licorice	They were randomly allocated to treatment either with deglycyrrhizinised licorice or placebo.	No differences were found between the treatment groups in the proportions with complete healing.	A randomized, double-blind, placebo-controlled trial.	[206]
12(Apparent Mineralocorticoid Excess)	Dietary Supplement: Licorice	Participants of the single-arm study will ingest licorice candy, and their blood, saliva, and urine samples will be collected.	No result posted	Interventional	NCT02939144
252	Extractum Liquiritiae Fluidum, 1 g diluted in 30 cc water, gargle the solution for 60 s without swallowing it starting preoperatively, 3 times a day until postoperative day 2.	Randomized allocation Licorice & Sugar water	Licorice gargling will be deemed better than sugar-water only if found non-inferior on both opioid consumption and pain score and superior on at least one of the two.	A Randomized, Double-blind Study	NCT02968823
60 (Oral lichen planus)	Licorice & Triamcinolone Acetonide	Triamcinolone mucoadhesive film & licorice mucoadhesive film	No result posted	Randomized by double-blind clinical trial	NCT02453503
63 (High Caries Risk Patients)	Arabic, Gum, Licorice Root, Chlorhexidine	Arabic gum and licorice root extracts compared to Chlorhexidine	No result posted	Randomized, Parallel Assignment,	NCT03684993
236 (Sore Throat)	Licorice Versus Sugar-water Gargle	Licorice solution, sugar solution	Licorice gargling halved the incidence of sore throat.	Randomized, Double-blind Comparison	[12]
66 (NAFLD)	2 g aqueous licorice root extract for 2 months	2 g aqueous licorice root extract and placebo	A significant drop in liver enzymes following administration of licorice root extract.	Double-blind randomized	[207]
60 (with SAE of CHB)	Tenofovir plus intravenous glycyrrhizin	Tenofovir plus intravenous glycyrrhizin and Tenofovir	Early introduction of glycyrrhizin can be safe and helpful for patients with SAE of CHB.	Randomized	[211]
57(hepatitis C patients)	Glycyrrhizin	Glycyrrhizin, or placebo	240 mg dose of glycyrrhizin thrice-weekly does not affect HCV-RNA levels and lowers the serum ALT during treatment, and it is well-tolerated and safe.	Randomized	[213]
69 (chronic hepatitis C)	Glycyrrhizin	Glycyrrhizin, or placebo	In individuals with chronic hepatitis C, glycyrrhizin therapy causes a substantial reduction in ALT. No major side effects were observed.	Randomized	[214]
1249 (chronic hepatitis with or without cirrhosis)	Intravenous glycyrrhizin injection	The treated and untreated group	Glycyrrhizin injection therapy significantly decreased the incidence of hepatocellular carcinoma.	Retrospective study	[217]
120 (dyspepsia either with peptic ulcer disease)	Licorice	Clarithromycin-based triple regimen, and study group that received licorice	Licorice enhances the eradication of *H. pylori*, particularly in the presence of peptic ulcer disease	Randomized controlled clinical trial	[219]
21 dental students	Glycyrrhizin	Glycyrrhizin and placebo	Glycyrrhizin has the potential to inhibit tooth plaque	Pilot study	[221]
